# Patient-Specific Preictal Pattern-Aware Epileptic Seizure Prediction with Federated Learning

**DOI:** 10.3390/s23146578

**Published:** 2023-07-21

**Authors:** Raghdah Saemaldahr, Mohammad Ilyas

**Affiliations:** 1 Department of Computer Science, Taibah University, Medina 42353, Saudi Arabia; 2 Department of Electrical Engineering and Computer Science, Florida Atlantic University, Boca Raton, FL 33431, USA

**Keywords:** epilepsy, seizure prediction, preictal, federated learning (FL), spiking encoder, graph convolutional neural network (GCNN), patient-specific personalization

## Abstract

Electroencephalography (EEG) signals are the primary source for discriminating the preictal from the interictal stage, enabling early warnings before the seizure onset. Epileptic siezure prediction systems face significant challenges due to data scarcity, diversity, and privacy. This paper proposes a three-tier architecture for epileptic seizure prediction associated with the Federated Learning (FL) model, which is able to achieve enhanced capability by utilizing a significant number of seizure patterns from globally distributed patients while maintaining data privacy. The determination of the preictal state is influenced by global and local model-assisted decision making by modeling the two-level edge layer. The Spiking Encoder (SE), integrated with the Graph Convolutional Neural Network (Spiking-GCNN), works as the local model trained using a bi-timescale approach. Each local model utilizes the aggregated seizure knowledge obtained from the different medical centers through FL and determines the preictal probability in the coarse-grained personalization. The Adaptive Neuro-Fuzzy Inference System (ANFIS) is utilized in fine-grained personalization to recognize epileptic seizure patients by examining the outcomes of the FL model, heart rate variability features, and patient-specific clinical features. Thus, the proposed approach achieved 96.33% sensitivity and 96.14% specificity when tested on the CHB-MIT EEG dataset when modeling was performed using the bi-timescale approach and Spiking-GCNN-based epileptic pattern learning. Moreover, the adoption of federated learning greatly assists the proposed system, yielding a 96.28% higher accuracy as a result of addressing data scarcity.

## 1. Introduction

**Figure 1:**
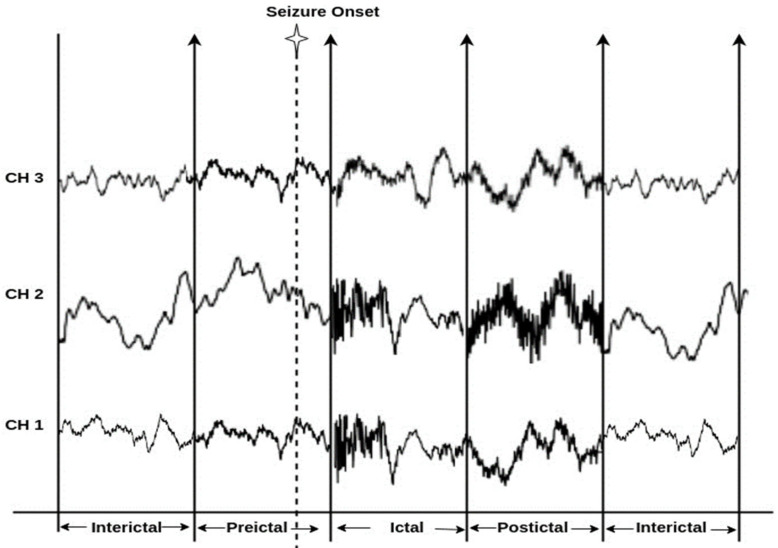
Diagram of epilepsy stages.

Epilepsy is a chronic neurological dysfunction syndrome characterized by repeated seizures induced due to irregular and excessive brain activity. Characteristics of seizure involve loss of consciousness, disruption of movement, and other cognitive malfunctions [1]. Epilepsy poses a severe disease burden; 70 million people are affected by epilepsy worldwide, according to the World Health Organization (WHO) survey, and about 20 million new epileptic patients are recorded each year [2]. Up to 70% of epileptic patients’ conditions are medically manageable using Anti-Epileptic Drugs (AED), whereas the conditions of 30% of people with epilepsy are unmanageable due to the unpredictability of their seizures [3]. Thus, epileptic seizure prediction has become extremely important to be able to save patients from seizures before they occur. Electroencephalograms (EEGs) record the brain’s electrical activity, serving as an analytical and diagnostic tool for epilepsy. EEGs are a widely used signal for measuring electrical, metabolic, or clinical changes in brain activity in order to observe the transition from the non-seizure state to the seizure state. EEG recordings of epileptic patients are categorized into multiple consecutive stages depending on the occurrence of seizures [4]. The preictal stage refers to that stage occurring before the onset of a seizure; the ictal stage refers to the phase while a seizure is occurring; the postictal stage refers to the period after a seizure; and finally, the interictal stage denotes the seizure-free period between the occurrence of two seizures [5]. Seizure onset [6] implies the actual generation of clinical seizures in the cortex. Figure 1 illustrates the epilepsy stages.

Epilepsy is a life-threatening disorder due to the occurrence of unexpected seizure episodes, creating a high psychological and social impact on epileptic patients. To promote quality of life, the prediction of epileptic seizures is necessary in order to control impending seizures [7]. It is essential to devise a potential siezure prediction system for patients who cannot be treated using medication. When processing epileptic EEG signals, seizure detection and prediction are modeled as classification tasks. Small seizure durations in EEG recordings indicate that the interictal state is comparatively longer than the ictal state. Hence, the seizure detection task attempts to differentiate the ictal state from the interictal state. In the subsequent identification of the preictal interval, seizure onset prediction critically supports the early medical diagnosis of epileptic seizures in patients.

Despite the abundance of epilepsy research activities, researchers have envisioned the possibility of performing accurate seizure prediction and interceding before the onset of seizure indications [8]. Conventional works utilize handcrafted features marked on EEG signals to locate the preictal state before seizure onset. However, manual feature extraction leads to inaccurate seizure prediction due to information loss and extends the warning time. Epileptic seizure prediction research works have employed various models, such as machine learning and signal processing [9]. Machine-learning-based seizure prediction and medical diagnosis have been accompanied by privacy concerns due to the considerable sensitivity of health data. In addition to the patient characteristics included in the health data, the processing of diagnosis results is also sensitive; hence, privacy becomes a major constraint. Even though research into machine-learning-based diagnosis has investigated different encryption methods, medical diagnosis systems are confronted with the challenge of low efficiency while needing to achieve high levels of privacy. The concept of Federated Learning (FL) [10] has emerged as a potential solution, and is able to overcome privacy issues while training machine learning models using the data of edge devices distributed worldwide.

Developing a high-performance model is crucial in order to provide a reliable, real-time medical diagnosis. The FL model protects users’ data by transferring only model parameters trained locally on the client device, instead of transferring clients’ data to the cloud. Owing to the shortage of clinical experts and the high cost of manual diagnosis, adopting FL improves the quality of healthcare service without an expensive diagnosis. In the FL, local model learning and decision making are performed by leveraging local data and global model knowledge, whereas the global shared model receives updates from distributed local models trained on various data. Hence, the main aim of the early epileptic seizure prediction system is fulfilled by the FL model while ensuring privacy, minimal latency, and minimal power consumption.

**Figure 2:**
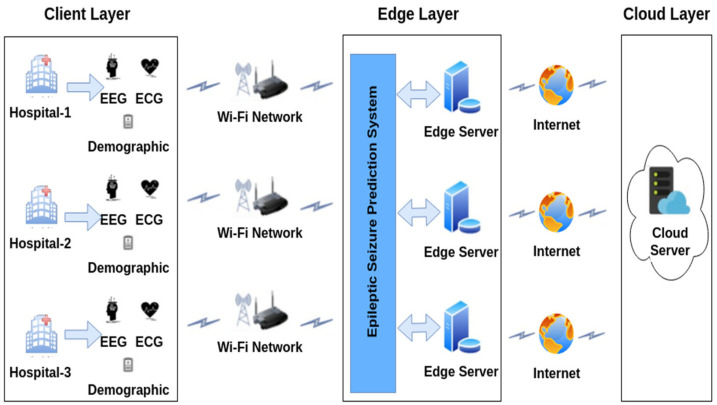
Pipeline of the proposed epileptic seizure prediction methodology.

Research works on conventional epileptic seizure prediction methods [11] have increasingly been applying Convolutional Neural Networks (CNNs); however, the high diversity and complexity of EEG signals deteriorate the prediction performance of this simple structure. Moreover, epileptic seizure prediction systems are faced with the computation of optimal or personalized preictal periods for the training set. Previous machine-learning-based seizure prediction approaches have investigated the use of fixed preictal intervals in the training samples. Patients have different preictal durations based on their characteristics before the seizure onset. Hence, examining patient-independent preictal periods is ineffective for accurate seizure prediction. Examining generalized seizure prediction involves classifying the preictal, interictal, and ictal states of EEG signals recorded from all of the scalp regions. Thus, as modeled in Figure 2, this work aims to build a patient-specific preictal period detection system for predicting seizure onset quickly and with high accuracy and privacy.

### 1.1. Contributions of This Work

In this work, a seizure prediction mechanism consisting of a three-tier architectureis proposed by applying federated learning and a hybrid deep learning model to accurately detect the preictal class. The notable contributions of this work are presented below:

- Contribution 1: Design of Seizure Prediction System
  ▪ Research Gap: Data scarcity, privacy, and manual assistance in real-time seizure prediction.
  ▪ Contribution: In this work, the preprocessing and classification stages are designed to take place in a two-level edge layer in the FL model instead of building local hospital models to ensure real-time prediction without manual interruption. Moreover, the postprocessing stage is automated using the Adaptive Neuro-Fuzzy Inference System (ANFIS).
  ▪ Achievement: As proof of principle in the FL, the data scarcity and privacy problems are resolved by utilizing the aggregated knowledge of seizure patterns from distributed EEG signals, and the design of a two-level edge layer mitigates manual interference.
- Contribution 2: Preprocessing and Classification
  ▪ Research Gap: Lack of preictal-aware learning process.
  ▪ Contribution: The proposed process employs a bi-timescale approach based on segment-aware training samples and models the layer-wise feature space of EEG signals to enhance the preictal state detection capability. In particular, in this work, a learning model is designed with a Spiking Encoder (SE) and a Graph Convolutional Neural Network (GCNN) to handle the diversity and complexity of the EEG signals. Furthermore, the local model utilizes global knowledge and distinguishes the preictal from the interictal state.
  ▪ Achievement: Global preictal-pattern-based coarse-grained personalization.
- Contribution 3: Postprocessing
  ▪ Research Gap: Need for patient-specific seizure prediction.
  ▪ Contribution: This work employs the ANFIS-PSO model for fine-grained preictal personalization and adaptively integrates the Heart Rate Variability (HRV) features and clinical features, along with the seizure probability obtained from the FL-assisted coarse-grained personalization to strengthen the patient-specific seizure onset prediction ability in the edge server.
  ▪ Achievement: More accurate seizure prediction.

### 1.2. Paper Organization

This paper, addressing seizure prediction, is structured as follows. Section 2 represents a literature review of research work performed on epileptic seizure prediction. The system model and problem formulation contemplated in the epileptic siezure prediction system proposed here are described in Section 3. Section 4 describes the proposed epileptic seizure prediction methodology. Section 5 presents the experimental evaluation with the experimental setup and datasets used in the proposed model and compares previous works. Finally, the conclusion and summary of the proposed approach are presented in Section 6.

## 2. Literature Review

In recent decades, machine learning models have gained significant attention for the prediction of the outcomes of healthcare services, including in disease prediction, pattern extraction, and decision making.

### 2.1. Deep-Learning-Based Epileptic Seizure Prediction Approaches

A patient-specific seizure prediction approach has been reported [12] adopting the CNN model to categorize the preictal stage on the basis of EEG and iEEG signals. Short-time Fourier transform is used to perform raw EEG data preprocessing with minimal effort in the feature engineering process. A seizure prediction framework [13] using Long Short-Term Memory Networks (LSTM) has been developed to analyze the preictal state on the basis of EEG signals. This LSTM-based siezure prediction system utilizes a broad range of feature extraction methods, namely, in the frequency and time domains, graph using theoretical measures and EEG correlation to impart solid ictal prediction performance. Another seizure prediction methodology has been presented [14] that was designed to distinguish between the preictal and interictal phases using CNN, and data equalization was performed in order to overcome the trial imbalance problem. This model utilized common spatial patterns and wavelet packet-based decomposition feature extractors to extract the temporal–frequency characteristics of EEG signals. Another epileptic seizure occurrence prediction model was presented [15] in which the multi-view CNN model was exploited to attain different views of EEG signals. This model acquires discriminative and adequate feature representations from EGG data using time- and frequency-domain methods. A seizure prediction approach [16] was developed in which LSTM was utilized to differentiate the preictal state from the interictal and ictal states on the basis of EEG signals. In this approach, a stacked Bi-LSTM network is built to achieve better seizure prediction performance before seizure onset. 

An end-to-end patient-specific model has been reported [17] in which CNN is employed to predict seizures before seizures. In this method, the CNN network is implemented using one-dimensional (1D) and two-dimensional (2D) kernels in the initial and final stages of the convolution and max-pooling layers to attain greater accuracy. The efficient seizure prediction approach [18] relies on CNN to extract features automatically and to classify the preictal and interictal segments of the EGG. In this approach, EEG channel optimization is conducted using the channel reduction technique in order to predict seizures on the basis of EEG signals. A patient-specific siezure prediction system has been reported [19] in which electrocardiogram (ECG) features, particularly the time and frequency features from the RR series, are examined by means of recurrence quantification analysis. Furthermore, it exploits the Support Vector Machine (SVM) model to classify preictal and interictal segments. To identify the preictal state from the EEG signals, the research reported in [20] investigated the HRV features of ECG signals while considering the frequency- and time-domain features for the recognition of each seizure. Early changes in the EEG and HRV features assist in characterizing the preictal and interictal states in drug-resistant epilepsy patients. The research reported in [21] developed an ANFIS-based seizure prediction system for patients affected by Parkinson’s disease. By modeling the ANFIS for the purpose of EEG signal analysis, the starting point of seizure onset could be detected, thus supporting real-time seizure prediction. However, performing real-time medical diagnosis on the basis of the examination of a single modality of EEG input data alone is ineffective due to the lack of exploration of different inputs.

### 2.2. Hybrid-Learning-Based Epileptic Seizure Prediction Approaches

A generalized deep learning framework has been reported [22] for seizure prediction employing the CNN-LSTM architecture. Initially, in this framework, Short-Time Fourier Transform (STFT) is applied to effectively carry out EEG signal preprocessing. Then, the features of sequential EEG segments are captured using spectral, spatial, and temporal methods, and the preictal EEG segments are distinguished from the interictal EEG segments, with high prediction performance. An effective patient-specific seizure forecasting method has been described [23] in which the Deep Convolutional Neural Network (DCNN) and Bidirectional LSTM (Bi-LSTM) models are employed to analyze the temporal and spatial features of raw EEG signals. Subsequently, this method enables a Deep Convolutional Auto-Encoder (DCAE)-model-based supervised learning method with transfer learning and channel selection to diminish the training time and computation load while predicting the seizure events. In the research work reported in [24], the EMD and DWT methods were employed to convert the raw EEG signals into the extracted features, which were then provided as the input to the classification models, specifically the Decision Tree, and their approach was evaluated using the Bonn EEG dataset. The epileptic seizure prediction system reported in [25] consisted of a method in which LSTM and CNN were combined, and a Long-term Recurrent Convolutional Network (LRCN) model was presented. The LRCN design was used to identify preictal segments by analyzing the spatial and temporal information in an EEG sequence belonging to the CHB-MIT dataset. A novel epileptic seizure prediction approach has been presented [26] in which a hybrid DenseNet-LSTM model is employed for forecasting patient-specific epileptic seizures. The hybrid DenseNet-LSTM model integrates the DCNN and LSTM networks. Furthermore, it applies Discrete Wavelet Transform (DWT) to the EEG signals, transforms them using CNN, and then classifies preictal and interictal states using LSTM. 

An epileptic seizure forecasting method has been developed [27] that is able to predict the preictal stage of seizure activity. This method encompasses a series of processes, including Empirical Mode Decomposition (EMD), for preprocessing EEG signals, Generative Adversarial Network (GAN), to overcome class imbalance issues, and CNN, to perform automated optimal feature extraction, while LSTM is exploited to robustly distinguish preictal and interictal segments. An epileptic EEG recognition approach [28] utilizes the improved residual network architecture to diagnose epileptic EEG, and different states of epileptic EEGs are automatically labeled. This improved residual network is an independent Recurrent Convolutional Neural Network (RCNN) composed of a One-Dimensional CNN to preprocess the essential features of EEG and an Independent Recurrent Neural Network (indRNN) to learn the correlations among EEG signal sequences and differentiate different ictal periods. Deep ensemble learning has been proposed [29] for epileptic seizure forecasting, where EMD is incorporated to remove noise and GAN to generate synthetic preictal stages. Subsequently, it exploits three-layered customized CNN to extract a comprehensive feature set and SVM, CNN, and LSTM in order to enable ensemble classifiers using Model-Agnostic Meta-Learning (MAML) to differentiate between preictal and interictal states. A new neuromorphic computing approach has been reported [30] in which the Gaussian random discrete encoder is employed to create spike sequences for the input EEG data. The combination of the energy-efficient SNN and CNN is able to perform seizure prediction by leveraging the potential advantages of each model. The seizure prediction approach [31] mitigates the need for higher computation consumption in information fusion by adopting a Graph CNN (GCN) that explores the graph structure of EEG signals. Designing a simple network architecture with node and edge features predicts seizures on the basis of scalp EEG signals. Despite this, the generalized graph structure can result in the medical misdiagnosis of individual patients, because the edge features in the graph are sensitive to differences among patients.

It can be concluded from the above literature analysis that there are different models for epileptic seizure prediction, and new solutions are emerging. However, there are several research directions in patient-specific preictal state detection leveraging early diagnosis that are not pioneering; therefore, several constraints must be resolved in order to achieve reliable seizure prediction in real time. For decision making in environments characterized by data scarcity, extracting other patients’ preictal information has not received adequate attention. Additionally, automated personalization on the basis of small EEG patterns without handcrafted features remains an emerging field of research. The fusion of multiple seizure-indicating features, such as EEG signals, ECG signals, and clinical records, requires further research for accurate seizure prediction. In deep learning, the handling of data scarcity and the preservation of privacy in small sensitive medical datasets have not been well studied. Hence, this work addresses these issues through the following attempt to produce a model for the task of epileptic seizure prediction.

## 3. Design of Epileptic Seizure Prediction

In the real world, the healthcare system highly demands Cloud computing technologies to combat the massive generation of voluminous data from the revolution of smart technologies. To avert the uploading and to store a tremendous amount of raw data on a centralized server, smart healthcare systems adopt the Federated Learning technique in a decentralized manner, ensuring the privacy of sensitive local data. 

### 3.1. System Model

This work presents an epileptic siezure prediction system that alerts patients and medical practitioners regarding the onset of epileptic seizures in distributed medical centers or hospitals.

**Figure 3:**
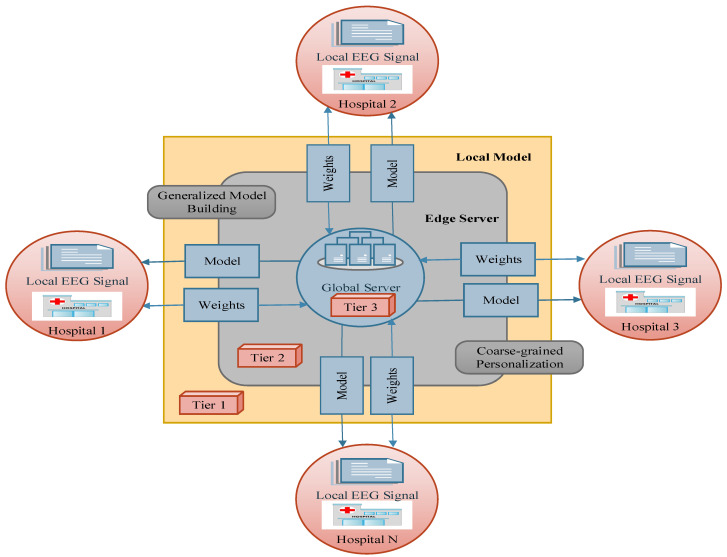
Three-tier architecture of the federated learning model in the proposed epileptic seizure prediction system.

With reference to [32], which was motivated by the analysis of ECG features, it is assumed that (i) the preictal interval search relies on state changes in EEG and ECG signals, and (ii) preictal state localization is influenced by ECG-related events that precede seizure onset in the EEG signals. Preictal period initialization does not strictly occur near seizure onset in each subject, and varies among patients; therefore, it is infeasible to employ a generalized consensus of the duration of the preictal period. To furnish medical services in a distributed client environment, the federated learning model adopts the benefits of using a centralized global server, and clients update their knowledge on the basis of the learning model parameters. The model-centric and cross-device FL model is employed to implement the proposed three-tier system. In federated learning, a model-centric approach refers to the storage of heterogeneous data at different local hospitals, while the learning model is fixed and centralized for training the different datasets. The cross-device refers to the receipt of data from different edge devices belonging to a single organization. To design FL-assisted epileptic seizure prediction, data from three different hospitals, using scenarios with the same feature space, were recorded from EEG channels, and different samples were obtained from across the three EEG datasets, regardless of the dimensions; these are referred to as sample-based FL and horizontal FL, respectively. In the distributed hospitals, each dataset comprises observations of brain activity monitored using different electrode placement systems at various sampling frequencies, and using different channels. In the FL design, decision making by the the local model is interrelated with the global model by aggregating the local model parameters from the different clients in order to perform model building on the cloud server. By modeling the FedAvg algorithm for aggregating the local model weights of different clients at each iteration, the proposed epileptic seizure prediction system repeatedly aggregates the model weights until convergence is achieved on the centralized global server as well as the local model outcomes. In FedAvg, the central server aggregates the parameters obtained from the distributed local models and distributes the global parameters to the clients. Federated learning greatly supports medical clients in the context of mobile healthcare, home healthcare, and hospital healthcare. This work predominantly intends to alert patients or caretakers before the onset of an epileptic seizure by identifying potential signs that can help to distinguish between the preictal and interictal states on the basis of EEG signal timeframes. Hence, the design of the proposed prediction model involves three major components in a three-tier architecture: the hospital as the client, the local model as the edge server, and the cloud as the global server. The proposed three-tier architecture is illustrated in Figure 3.

Tier 1 in the three-tier architecture gathers EEG signals from a diversity of patients in distributed hospitals. In the hospitals, EEG signals are recorded following the placement of non-invasive electrodes using a 10–20-electrode placement system and different mobile EEG systems like headsets, mobile EEG caps, and saline-based electrodes. Tier 2 is responsible for utilizing the data collected from the hospitals and building the local model for each hospital in the corresponding edge server, rather than building the local models in the hospitals themselves. Tier 3 remotely builds a global model on the basis of the knowledge of local models in order to discriminate between different states within the EEG timeframes.

Client (Hospital): The proposed system contemplates a horizontal FL model, which utilizes the distributed datasets with the same feature space across all clients, implying that hospital 1 and hospital 2 have the same feature model in different dimensions, with different channels as the columns. The region of the hospital or medical center is modeled as tier 1 in the three-tier epileptic seizure prediction system architecture.

Local Model (Edge Server): In the FL model, the edge server is critical in providing computation and data storage functions closer to the clients, thereby enhancing network availability while ensuring minimal latency. Due to the potential advantages of the edge server, the transmission time is comparatively low compared with transmitting to the cloud. Hence, request latency is minimized. Moreover, due to there being comparatively less network traffic in each edge server within the server’s coverage area, the network availability remains high at the time of requests compared to network availability in the cloud. Time-sensitive healthcare applications greatly benefit from edge computing capabilities compared to huge data generation. On the basis of the distributed EEG signals obtained from various hospitals, the FL model builds a local model for each hospital and builds a global model without transferring the local data to the cloud or other clients, referring to the ‘Model-Centric’ FL concept.

Global Model (Cloud): Cloud computing infrastructure acts as the global model for remote processing across the sensitive data belonging to hospitals distributed in the horizontal FL. This involves the aggregation of global knowledge based on the different preictal patterns obtained from the different local models using FedAvg.

**Figure 4:**
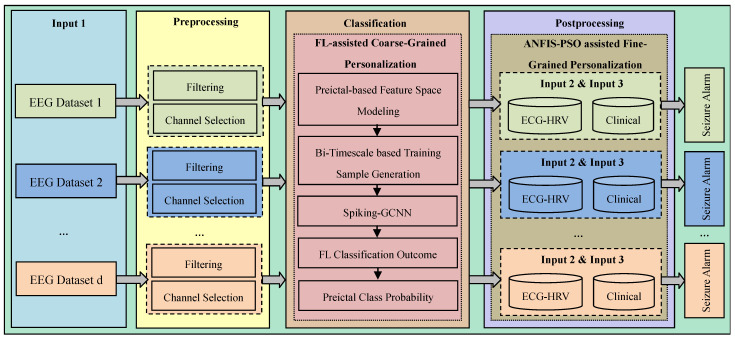
Process diagram of the proposed seizure prediction system.

As discussed above, in this work, a three-tier architecture is designed. Furthermore, in this work, the full epileptic seizure prediction procedure is divided into three stages: preprocessing, classification, and postprocessing, as outlined in Figure 4. In the proposed system, the preprocessing and classification processes are modeled in the edge server with reference to the FL concept. In the classification phase, FL-assisted coarse-grained personalization is framed as a binary classification problem in which the preictal and interictal classes are to be differentiated. During postprocessing, the proposed approach only utilizes the preictal class probability after obtaining the outcome from the coarse-grained binary classification model. In particular, postprocessing involves ANFIS-based decision making, which is executed in the edge server’s first layer to mitigate the client’s computational burden. 

### 3.2. Problem Formulation

In the biomedical field, disease diagnosis systems have often encountered challenges in predicting epileptic seizure patterns from EEG signals related to data scarcity, diversity, and privacy. In this work, two major research constraints are considered in the epileptic seizure prediction system: model generalization and preictal state identification.

**Definition** **1** **(Model** **Generalization).** *Owing to the patient’s pattern diversity, the patient-specific epileptic siezure prediction systems have been predominantly focused on the previous works [19,33] rather than patient-independent decision making. The model generalization is essential to overcome several shortcomings of epileptic seizure prediction research, involving the scarcity issue of preictal samples and the computational burden in frequently training the models for every patient.*

(1) $$\underset{i}{\min}\left(\frac{1}{S}\sum_{i=1}^{S}L_{i}\left(\theta ,\phi \right)\right)$$

*During epileptic seizure prediction, the generalized model-assisted prediction capability maximizes the first research objective by minimizing loss among the number ‘S’ of patients (i). In Equation (1),* θ and ϕ *refer to the learning model’s actual and predicted value parameters for the computation of loss.*

**Definition** **2** **(Preictal** **State** **Identification).** *The numerous developments in EEG signal processing research notwithstanding, seizures are unpredictable due to the lack of medical theory related to proving the prediction results. This epileptic siezure prediction system intends to differentiate between the preictal and interictal states, considered as a binary classification problem, as the second research objective. In the sequence of time series EEG signals, the preictal state is the period immediately before the seizure onset* [1,7]. *In the preictal state, the predictive probability of seizure is the same as throughout the sample segments, which is not intuitively acceptable, due to the lack of discrimination between the preictal and seizure state over different periods. Hence, the minimization of the loss across the number ‘V’ of EEG segments (r) within a patient ‘i’*.

(2) $$\underset{r}{\min}\left(\frac{1}{V}\sum_{r=1}^{V}L_{r}\left(\theta ,\phi \right)\right)$$

*In Equation (2), actual and predicted values are computed for each EEG segment (r) in the learning model. Quantifying the changes in the seizure pattern in the EEG signals and modeling a prediction system to determine the epileptogenic transient changes is challenging. Quantitative EEG measures involve the periodic analysis of frequency patterns over the course of the sequence of EEG signals to detect seizure transitions. The length of the preictal state, prior to seizure onset, varies between patients from a few minutes to hours. In addition, the selection of an inaccurate preictal duration drastically affects the prediction results. Hence, preictal period selection should be performed in a patient-specific manner to avert an increase in the number of false predictions and a decrease in prediction sensitivity due to fluctuations in the preictal period. Thus, this work aims to maintain the trade-off between model generalization and patient-specific preictal period selection or preictal probability identification without compromising the prediction performance in the epileptic seizure prediction system.*

## 4. Proposed Epileptic Seizure Prediction Methodology

With the aim of predicting epileptic seizures, this work focuses on designing a federated learning process for local model building, generalized model building, global knowledge aggregation, global preictal knowledge unification, and distinct preictal period modeling. 

**Figure 5:**
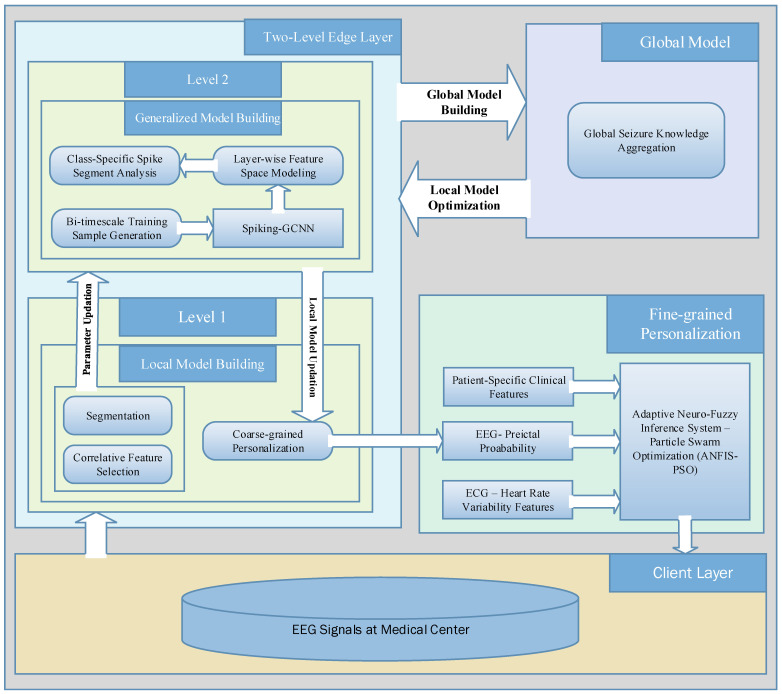
The proposed epileptic seizure prediction methodology.

Figure 5 depicts the proposed epileptic seizure prediction approach and the FL-assisted process. The edge server is responsible for automating epileptic seizure prediction with the assistance of the cloud, which involves EEG preprocessing, generalized and distinct feature extraction, and personalized federated learning-based seizure prediction. In other words, in the proposed approach, a two-level edge layer is designed, where the data-aware process is incorporated in level 1, the lower layer in the edge layer, and the model-aware process in level 2, the upper layer in the edge layer. In the two-level edge layer, the data-aware process involves filtering, correlative feature selection, segmentation, and patient-specific preictal modeling, whereas the model-aware process involves generalized model building and distinct preictal period modeling. The computational steps in the lower and upper layers are subsequently performed in the lower and upper layers. Conversely, even though patient-specific preictal modeling is designed as being part of the lower level of the edge layer, it relies on the outcomes of distinct preictal period modeling, which is performed in the upper level of the edge layer. 

### 4.1. EEG-Signal-Based Distributed Local Model Building

In this section, the design of the distributed local models on edge servers on the basis of EEG signals obtained, while adopting the federated learning model, from hospitals or medical centers as clients.

#### EEG Signal Preprocessing

In seizure prediction, scalp EEG signals are paramount to recognizing the different ictal segments, and are recorded by electrodes placed on the individual’s scalp. In the proposed system, EEG signal preprocessing is imperative in order to remove artifacts and noise. Recently, signal processing techniques have enabled the system to automatically identify and remove artifacts in EEG-based seizure prediction systems [34,35]. To model the end-to-end automatic epileptic seizure prediction system, in the proposed prediction approach, several preprocessing procedures are utilized alone, without the requirement of human interference for feature extraction. As a result, filtering, artifact removal, and correlative feature or channel selection are conducted in the preprocessing step.

(i) Filtering and Artifact Removal: In the proposed prediction model, the window and Butterworth filter methods are applied to filter the noise. When preprocessing EEG recordings, the second-order Butterworth bandpass filter is critical in examining and removing artifacts [36]. Due to the importance of providing a linear frequency for bandpass filtering, the Butterworth bandpass filter is employed as an EEG signal preprocessing filter. Moreover, preprocessing involves normalizing and chunking the signals into fixed lengths. In high-dimensional data, EEG signal values vary greatly from channel to channel, which becomes critical during model training. Hence, normalization is essential for EEG signal processing, which constitutes the processing of structured data in the learning model. Moreover, the input EEG signals are divided into minimal periods of 1 s samples, corresponding to the sliding window length, based on the sampling rate of EEG recordings to precisely normalize the EEG signals per second rather than normalizing them with reference to the entire timeframe.
(ii) Correlative Channel Selection: To accurately predict seizures, in the proposed approach, a minimal number of channels is selected, because all channels have equal significance in epileptic seizure prediction. Investigating the correlation between different channels means that examining the role of the interaction among brain regions in modulating the epileptic seizure activity becomes a potentially crucial standard for the prediction of seizure onset [37]. The channel reduction in the seizure prediction system ensures the potential advantages of minimal energy consumption, reduction in overfitting rate, and increased time efficiency [38,39]. In this correlative channel reduction scheme, in the proposed approach, the significance of each channel’s contribution to the classification outcome is evaluated through the wrapper feature selection method [40]. If any channel does not result in an improvement in accuracy, that channel is ignored in the proposed approach, which does not reduce performance in the future. To perform the correlative channel selection, the Taguchi-method-based optimization [41] is adopted in the proposed system, which provides potential information using the minimum number of experiments based on the Design of Experiment (DoE) concept. In channel reduction, iteration continues until the model’s accuracy decreases when removing any further channels from the final set. Thus, the proposed approach efficiently selects the EEG channels for epileptic seizure prediction.

### 4.2. Federated-Learning-Based Generalized Model Construction

In the biomedical community, resolving the data scarcity constraint is becoming a major challenge in accurately diagnosing patients, due to inadequate data availability regarding epileptic seizures for each patient. In contrast, deep learning models require large amounts of training data or patterns to perform decision making. To overcome this obstacle, the proposed approach adopts federated learning for model generalization, whcih supports the utilization of the influence of the epileptic seizure patterns of a diversity of patients. In the edge environment, model generalization is essential for predicting seizure onset when considering segment-aware training sample generation and spike sequence-aware pattern modeling, in addition to only utilizing the model parameters from multiple clients.

#### 4.2.1. Segment-Aware Training Sample Generation

The key factor in the proposed system is the differentiation between the preictal and interictal states. Hence, the segmentation process necessitates the recognition of standard state transitions, as provided by clinical experts. The statistical features of non-stationary EEG signals vary within a given time interval. Accordingly, based on the data distribution, in the proposed approach, long sequences are divided into EEG segments of short duration with or without overlapping EEG signals. Thus, sensing EEG signals with shorter durations has benefits in the form of minimizing requirements in terms of computational power and storage requirements, as well as low transmission bandwidth. In the proposed epileptic seizure prediction system, the input EEG samples are segmented into different timescale-based EEG samples. Segmentation is performed based on the timescales 1 s, 2 s, 4 s, and 8 s [42,43]. Initially, the input EEG signals are segmented into four different timescales in the process of segmentation, and then, the bi-timescale approach is performed for decision making. In the bi-timescale approach, two segments are selected from among four segments for each dataset, and binary classification decisions are made jointly in during coarse-grained decision making.

Bi-Timescale Approach: EEG signals are time dependent; hence, in the proposed approach, the segmentation and learning of temporal signals are performed with the assistance of bi-timescale modeling, which assists in comprehending inherent relationships in the time domain. EEG signals are likely to comprise different types of potential information in various timescales. Hence, investigating four timescales and selecting two optimal timescales results in a significant improvement in performance for different EEG datasets. Finally, two types of timescale information are concatenated for decision making in the GCNN model.

**Figure 6:**
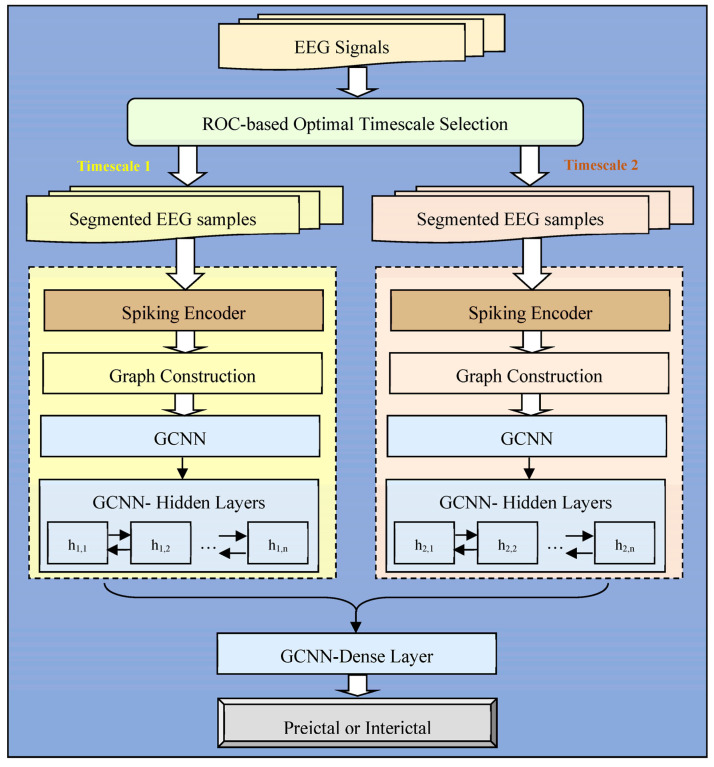
Bi-timescale approach based on segmented samples learning.

Due to the collection of EEG signals over long periods, the segmentation of the signal is essential for performing analysis by modeling representative forms of periodic and non-stationary EEG signals as smaller and mutually exclusive segments. Different EEG signals are gathered at different sampling rates in the distributed environment. Hence, signal segmentation is critical for recognizing preictal patterns. Accordingly, the selection of optimal signal partitioning is carried out based on EEG time segments of 1 s, 2 s, 4 s, and 8 s, employing sliding window concept, in order to determine any two different EEG segments that impact the accurate preictal state detection in the corresponding dataset, with respect to the bi-timescale-based learning process, depicted in Figure 6. In previous research works in which EEG segmentation was performed [42,43], the durations of EEG segments were widely modeled as either 1 s, 2 s, 4 s, or 8 s; hence, this work considers all four segmentation lengths in order to optimally select the appropriate length for each dataset by means of the bi-timescale approach. The proposed system adaptively selects the most highly influencing EEG segment length for each dataset in order to uniquely examine the seizure patterns in the different distributed EEG datasets, which is determined by means of ROC curve analysis. The significance of ROC-curve-based examination lies in its measurement of the relationship between true positives and false positives in each dataset, facilitating the assigning of the optimal segment duration for the corresponding dataset. Thus, the optimal EEG segment is found for a single dataset in which the segment is optimal in all channels, which is then taken into account during the training of the learning model.

In the EEG signals, modeling the training samples enforces the learning behavior of deep learning to distinguish between preictal and interictal patterns. With the assistance of ROC analysis, the optimal selection of EEG segments, accomplished using the bi-timescale approach, from the multiple timescales plays a vital role in the decision-making process, because each timescale conveys different potential information to the learning model. To generate the training samples, the segments are formed for both the preictal and interictal classes of the EEG signal using a sliding window, with and without overlap, respectively, based on the ratio of samples in the classes to ensure balanced operation, with sliding window length being based on the segmentation timescales. The aforementioned 1 s, 2 s, 4 s, and 8 s timescale-based EEG segmentation is performed using the sliding window concept. The interictal state is regarded as belonging to the non-seizure EEG recordings, whereas the preictal state is sensitive to the duration before the seizure. Instead of modeling only one timescale of EEG data as a single training sample, this research considers additional timescale signal durations as a single training sample in order to mitigate the noise effect in the training knowledge. As a result, training set generation is influenced by the number of segments in the preictal and interictal parts when performing balanced data modeling with the aim of successfully recognizing seizure state transitions.

#### 4.2.2. Spike Sequence-Aware Feature Space Modeling and Global Modeling

According to the neuroimaging concept of epilepsy [7], the interictal state corresponds to the seizure-free period, and the preictal state describes the pre-seizure period in the time series of EEG signals. The length of the preictal period varies among patients with different characteristics, and no reference standard is available to conclude any particular preictal period. Hence, with the assistance of FL, in the proposed approach, the knowledge of different preictal patterns, learned from different preictal datasets, is incorporated, which facilitates conclusions about generalized preictal patterns on the basis of global model weights. The interictal period is the duration belonging to neither the preictal nor the ictal period in the sequence of EEG signals. To date, epileptic seizure prediction research works have employed CNN and RNN models [44,45] for classifying the high-dimensional preictal and interictal EEG patterns in the spatial and temporal domains; however, the conversion of EEG signals into a Euclidean grid structure causes the results to suffer from a loss of adjacent spatial information. Hence, the proposed approach exploits the Graph Convolutional Neural Network (GCNN), following the use of a spiking encoder, which consumes minimal computational and storage resource across the channels after feature extraction. In the proposed epileptic seizure prediction system, the main objective of applying a spiking encoder is to encode and represent the input EEG signals as the spike-aware sequence representation. Due to the addition of the matrix in the computation of the Spike Neural Network (SNN) model [46,47], instead of the multiplication process, the Spiking Encoder (SE) becomes energy efficient [48]. Moreover, the SE is able to model dynamic modes of network operation by encoding the temporal information in the signals. As a result, the SE is more computationally and energy efficient.

(i) Spiking Encoder-Based Feature Space Modeling

In the EEG signals, in the proposed system, the spikes are detected throughout the duration of the EEG for all channels. Hence, in the proposed system, the deep neural network is designed with a spiking encoder, a Graph Convolutional Neural Network model, and weight mapping in order to predict seizures. In this approach, the spiking-GCNN model is built for two sets of training samples generated from optimally selected EEG segment durations, until the hidden layer representation of the GCNN model is achieved. In the bi-timescale approach, the representations obtained from the two optimally selected timescales are concatenated, and they are provided as the input to the dense layer of the spiking-GCNN model, as illustrated in Figure 6. Instead of extracting the statistical features using time-domain-based signal processing methods for graph construction, in the proposed approach, the features are examined automatically by the spiking neural network, which infers the reflection in the shape features in the time domain, including deviations in the signal amplitude, changes in the slope, and differences in the number of spikes. In the hybrid Spiking-GCNN model, the weights are trained for the CNN model associated with the energy- and computationally efficient Spiking encoder model mapping, in which the SE represents the EEG data as spike sequences. To precisely generate the spike representation, the proposed system models the spiking encoder using the surrogate gradient method, which enables backpropagation in the feed-forward neural network for the discrete nature of spikes. To build the spiking encoder, the ‘up’ and ‘down’ threshold parameters are determined for each segmented timestep in the sequence of EEG signals. Instead of creating a random matrix for the input timesteps, in the proposed approach, a segment-aware matrix is built to model signal amplitude values for the segmented timesteps and channels. By generating a segment-aware matrix, the encoder model compares every signal value in the input data with the mean value obtained from the mean of the ‘up’ and ‘down’ threshold points. If the mean value is greater, the spiked value of a particular signal will be equal to ‘0’; otherwise, the spiked value will be equal to ‘1’. Moreover, the encoding representation of the input sample in each segment relies on the number of spikes per sample and the spike average per sample duration.

(3) $$R{\left(S\right)}_{t}=\left\{\begin{array}{c} {pr}_{seiz}=\mathrm{High},\;\;\mathrm{if}\;\frac{1}{m}\sum_{j\in m}{\left|Sp\right|}_{t}^{q}<{\left|\mathrm{Sp}\right|}_{t}^{q}\&{avg\left|D_{Sp}\right|}_{t}^{q}>{\left|D_{Sp}\right|}_{t}^{q}\\ {pr}_{seiz}=\mathrm{Low},\;\;\;\;\;\;\;\;\;\;\;\;\;\;\;\;\;\;\;\;\;\;\;\;\;\;\;\;\;\;\;\;\;\;\;\;\;\;\;\mathrm{Otherwise} \end{array}\right.$$

The formulation of Equation (3) is based on the strategy whereby high numbers of spikes and spikes with a comparatively minimal duration indicate that a segmented timestep has a high probability of seizure onset (${pr}_{seiz}$). Consequently, the representation of the signals in the preictal class has vector values with higher weights than in the interictal class. In Equation (3), ${\left|Sp\right|}_{t}^{q}$ and ${\left|D_{Sp}\right|}_{t}^{q}$ denote the number of spikes in segmented timestep ‘t’ in the qth channel and the duration of the spikes in segmented timestep ‘t’ in the qth channel, respectively. ‘m’ refers to the total number of channels and $\mathrm{avg}{\left|D_{Sp}\right|}_{t}^{q}$ refers to the average duration of spikes occurring in each segment. By applying the ‘AND’ logical operator between the spikes computed from the spiking encoder and the seizure probability assigned by Equation (3), i.e., $E_{t}^{S}\cdot R{\left(S\right)}_{t},$ in the proposed approach, the spike sequences are fine tuned. $E_{t}^{S}$ refers to the encoded value of the input signal (S) in segmented timestep ‘t’ obtained using the spiking encoder. Thus, the spiking encoder generates a high-level abstraction of the input signals with the influence of the weight update using surrogate gradient-descent-based backpropagation, and the GCNN differentiates between the preictal and interictal states through the modeling of the node and edge parameters in the graph structure.

In the proposed method, the GCNN is trained on the input data to obtain weights, and the weights are mapped using the spike sequences transformed by the spiking encoder to predict seizures on the basis of the signals. In the proposed approach, a graph is built, $G=\left(V,E\right)$, to provide the encoded knowledge for the GCNN model, in which $V=\left\{v_{1},v_{2},\ldots ,v_{m}\right\}\;,\;$ referring to a set of ‘m’ channels, and ‘E’ denotes the connectivity between the channel electrodes on the patient’s brain. In the proposed system, the node features are the vector representations obtained from the fine-tuned spike sequences. The adjacency matrix, $A\in R^{m\times m}$ comprises the edge features of the relationship between the channel values in the preprocessed EEG signals. To address the problems of numerical instability, dispersion, and gradient explosion over multiple iterations, renormalization is applied, and the layer-wise propagation rule is employed in the spiking-GCNN, as described in [31]. The bi-timescale-approach-based spiking-GCNN modeling achieves layer-wise propagation. Instead of examining the node and edge relations on the basis of the graph structure alone, modeling the representation matrix resulting from the spiking encoderin each layer facilitates the accurate recognition of the inherent relationships in each class.

(4) $$f\left(M^{L},A\right)=\sigma \left(\left({\tilde{D}}^{-\frac{1}{2}}A{\tilde{D}}^{-\frac{1}{2}}\right)M^{L}{{R\left(S\right)}^{L}W}^{L}\right)$$

In Equation (4), $M^{L},{R\left(S\right)}^{L}$, and $W^{L}$ refer to the activation matrix, the representation matrix obtained from the spiking encoder, and the layer-specific weight matrix in the L^th^ layer, respectively. ‘A’ and ‘$\tilde{D}$’ denote the adjacency matrix and renormalized degree matrix of the graphs, respectively. $\sigma \left(\cdot \right)$ denotes an activation function in the GCNN model.

(ii) Global Knowledge Aggregation

The deployment of intelligent models necessitates periodic training and updating, placing a burden on medical practitioners or experts, who are required to generate the annotated labels for the massive volume of patients at any given time. Hence, federated learning mitigates this constraint across hospitals during the peak volume season, enabling medical centers to download and exploit the most up-to-date model for epilepsy diagnosis. Accordingly, the adoption of federated learning leverages the ability of the proposed system to utilize other patients’ epileptic patterns to improve seizure prediction performance. Even though variations exist across patients, there is a root-cause pattern similarity of epilepsy disease appearing between patients. Accordingly, the proposed prediction system trains a model whereby a global model, which is trained on other patients’ data, is used to update the local model with globally trained parameters. Adopting the global model parameters in each local model assists in the accurate prediction of epileptic seizures without jeopardizing performance.

During the training stage in generalized federated learning, in the proposed approach, a global model is built based on the loss function of the prediction model ($L_{PM}$), computed across all patients or subjects (S). In the seizure prediction system, the different states (C) refer to the preictal and interictal states observed in subjects ‘S’ in different local medical centers. Let $X_{s}=\left\{x_{1}^{s},x_{2}^{s},\ldots ,x_{T_{S}}^{s}\right\}$, denoting a set of EEG samples from the subject ‘S’, where $T_{S}$ denotes the number of training samples. Let $Y_{s}=\left\{y_{1}^{s},y_{2}^{s},\ldots ,y_{T_{S}}^{s}\right\}$, denoting a set of labels from the state ‘C’. To predict epileptic seizure, in the proposed system, the weights are mapped to the softmax-based probabilistic distribution $\left(p_{i}^{s}\right)$.

(5) $$L_{PM}=-\sum_{i=1}^{S}\sum_{j=1}^{T_{S}}\sum_{k=1}^{C}\omega \left(y_{j}^{i}=C\right)\log\left({\left(p_{i}^{s}\right)}_{C}\right)$$

As formulated in Equation (5), ω(.) will be equal to 1 if there is equality between the actual and predicted outcomes; otherwise, it will be equal to zero. In Equation (5), i, j, and k refer to the patient or subject, the training sample, and the class, respectively. $y_{j}^{i}$ denotes the label of the jth training sample of the ith subject. Thus, the proposed approach aggregates the preictal segment-based knowledge with the appropriate global model parameters.

### 4.3. Coarse-Grained Personalization with Optimal Trade-Off

In the proposed system, each local model receives the weights from the aggregated global server and is updated to perform decision making on its local EEG dataset. This is referred to as coarse-grained personalization, and is accomplished by the FL model. Furthermore, it maintains an optimal trade-off between the generalized and patient-specific personalized models. With the objective of iteratively updating the local model using the shared weights, the proposed approach measures the divergence between the probabilistic distribution of the generalized global model ($P_{G}$) and the personalized local model ($P_{P}$) using Bregman divergence [49]. In the proposed system, the Bregman divergence is computed based on the asymmetric measurement of logistic loss. The probability of the global model ($\bar{P_{G}}$) in the proposed approach is normalized by analyzing the impact of the probability of the seizure computed from the outcome of the fine-tuned spiking encoder, as shown in Equation (6). The reason behind this is that the personalized model needs to be updated for a higher probability of seizure onset in the number of ‘High-${Pr}_{seiz}$’ samples (${N(Pr}_{seiz}=High)$) for the corresponding local dataset, as formulated in Equation (7).

(6) $$L_{div}\left(P_{G},P_{P}\right)=\bar{P_{G}}\log\left(\frac{\bar{P_{G}}}{P_{P}}\right)+\left(1-\bar{P_{G}}\right)\log\left(\frac{\left(1-\bar{P_{G}}\right)}{\left(1-P_{P}\right)}\right)$$

(7) $$where\;\bar{P_{G}}=\left\{\begin{array}{c} P_{G},\;\mathrm{if}\;{N(Pr}_{seiz}=\mathrm{High})>N({Pr}_{seiz}=\mathrm{Low})\\ \log P_{G},\;\mathrm{Otherwise} \end{array}\right.$$

In the proposed approach, the loss of the client data is computed by contemplating the prediction loss ($L_{Pred}$) and the divergence loss ($L_{div}\left(P_{G},P_{P}\right)$) with two constant weights ($w_{1}$, $w_{2}$), respectively. As modeled in Equations (6) and (8), the loss divergence and loss are computed for the global-to-local and personalized local models, respectively.

(8) $$L_{P}={{w_{1}\times L}_{Pred}+w_{2}\times L}_{div}\left(P_{G},P_{P}\right)$$

Instead of sharing the sensitive raw medical information of the patients and the network structure with the global model, the proposed approach shares only the weights derived in the local model. A central hub in federated learning coordinates the learning process of all of the clients by means of global modeling and ensures improved accuracy comapred to clients’ local models, assuming that the data distribution of all clients is similar. In the proposed system, the training process of local models is carried out on an edge server for each hospital, and each client tests their samples while contributing to the global model and the local model, which are maintained by the cloud and the edge server, respectively. In consequence, each client benefits from the knowledge obtained by the global deep learning model, thus compensating for the fact that its own knowledge was learned from a minimal amount of training data in its corresponding hospital. To determine the number of iterations in federated learning, the early stopping method is adopted as the termination criterion, where the model is retained on the basis of the improved performance after a fixed number of epochs. Consequently, the proposed approach differentiates the preictal state from the interictal state, and preserves the patients’ privacy during the training process. Thus, the proposed approach, to a significant degree, maintains the trade-off between model generalization and personalized preictal detection in the epileptic seizure prediction system.

### 4.4. Distinct Preictal State Modeling for Seizure Prediction

**Figure 7:**
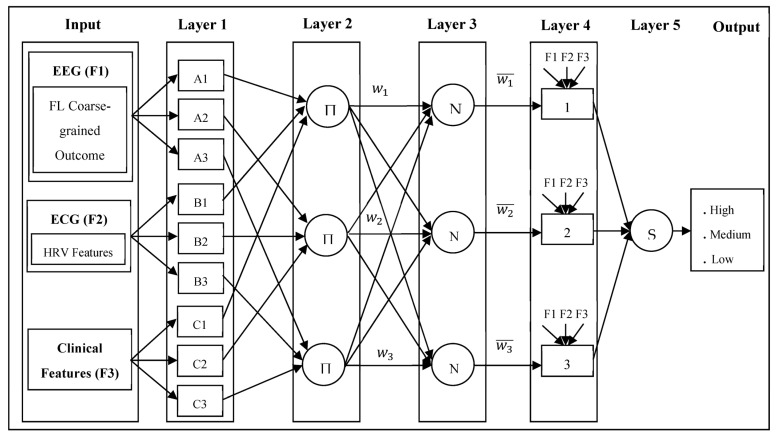
Three-input and three-membership-function ANFIS architecture for fine-grained personalization.

In the proposed system, postprocessing includes the performance of distinct preictal period modeling, which is highly correlated with patient-specific clinical features, followed by the coarse-grained personalization obtained using FL-assisted decision making. The proposed approach utilizes several potential clinical features and the Heart Rate Variability (HRV) of each epilepsy patient with the assistance of ANFIS-PSO [50,51] on the edge server to perform fine-grained personalization. Figure 7 illustrates the ANFIS architecture used in the proposed epileptic seizure prediction system.

#### ANFIS-PSO-Based Fine-Grained Preictal Personalization

The prediction of seizures and the diagnosis of epilepsy rely on both the electrophysiological and clinical data of each patient, in addition to the generalized global knowledge aggregated from the distributed medical centers. The correlation between the clinical features of a seizure and any electrical abnormalities identified in the input EEG records and ECG signals facilitates the fine-grained personalization of preictal state identification. The seizure onset time varies among patients, and its recognition depends on several seizure-related behavior changes. To avert confusion with respect to seizure onset identification, additional clinical factors are exploited, and the precise determination of seizure onset is enforced through patient-specific modeling of the preictal duration. The actual start of the preictal period varies from patient to patient. Hence, the proposed approach utilizes the ANFIS-PSO model and examines the clinical features, including the patient’s age, gender, family history, etiology, and Heart Rate Variability (HRV) characteristics [52] in the time domain and frequency domain, as well as non-linear features.

For testing purposes, in this work, such HRV features are synthetically modeled with reference to several previous research works. Even though these works [53,54] had different objectives, the ranges of several HRV features were determined based on observations obtained while monitoring normal and epileptic patient categories in different states. Hence, the ranges are analyzed in combination for epileptic patients on the basis of the minimal and maximal changes in the observations of each feature, and the observed ranges are divided into three classes for the proposed algorithm based on the HRV feature value transitions obtained from two references corresponding to different states.

The ANFIS model forces the proposed system to build a set of fuzzy if–then rules based on the membership functions by modeling input–output pairs. Combining fuzzy if–then rules, the fuzzy inference system, the adaptive structure, and the adaptive learning rule in the ANFIS ensures improved outcome quality compared to fuzzy logic. The integration of PSO with the ANFIS model facilitates the optimal selection of learners and ensures computationally efficient and optimal decision making [55,56]. The design of the fine-grained personalization of the preictal period with the aim of performing epileptic seizure prediction adopts ANFIS modeling with nine input parameters, which are obtained from three different inputs: EEG, ECG, and the patient’s clinical records. The membership function parameter iscalculated based on the embedded relation between the input data and the training dataset output.

In the ANFIS-PSO, tuning the membership function parameters relies on backpropagation. The learning process continues the evolution of membership functions until the target error is reached. In the proposed system, premise and consequence parameters in the membership function are updated based on the Mean Absolute Error (MAE) during backpropagation. Defining the target error for the ANFIS-PSO model is dependent on the minimal error across all of the iterations compared to the selection outcome of the learners using the PSO model. Moreover, the PSO-based classification error validation enforces the tuning of ANFIS parameters in terms of the number and shape of the input membership function, in which the selection of the best learner relies heavily on the higher fitness score of the PSO algorithm. To reduce the output error of the fuzzy inference system, the optimization process involves adjusting the network parameters and weights and interpolating the fuzzy membership function computation for a set of variables. In the proposed system, the Sugeno-type fuzzy inference system is modeled to examine the mapping relations between the input and output data values and to optimally compute the membership function. The antecedent and consequent parameters perform fuzzy reasoning on the basis of the linguistic variables and the outcome of the target variables on the basis of if–then rules with logic ‘AND’ operations, respectively. The triangular membership logic-based membership function is modeled using the reference for each variable. Thus, the initial fuzzy sets with input variables for the proposed ANFIS-PSO decision-making process are presented in Table 1. In the proposed design, the consequent is modeled as the seizure risk level based on the potential observation of preictal probability in EEG and HRV features.

In addition to the EEG analysis, by examining the HRV features of ECG signals and clinical features, in the proposed approach, the probability of epileptic seizure is computed for all samples, with the preictal probability being predicted on the basis of the FL-assisted coarse-grained personalization. The system categorizes a set of patients (k), according to three preictal period intervals on the basis of the influence of the HRV and clinical features of the corresponding patient in order to determine the likelihood of the occurrence of an epileptic seizure. In ECG signals, the R–R interval refers to the duration between two successive R waves in the QRS signal. Several HRV features of the ECG signals considered in this work are described in Table 2.

While analyzing the HRV features, in this work, we additionally focus on modeling high-, medium-, and low-risk preictal periods in epileptic patients based on the criteria shown in Equation (9) throughout the sequence of the ECG signals. Chinges in the HRV ($\hat{{HRV}_{i}}$) are recognized as indicating the transition on the basis of an inherent examination of the triggering state of different preictal states.

(9) $$P_{i}^{x}=\left\{\begin{array}{c} P_{k}^{1}\;,\;\mathrm{if}\;\hat{{HRV}_{i}}==\alpha \\ P_{k}^{2}\;,\;\mathrm{if}\;\hat{{HRV}_{i}}==\beta \\ P_{k}^{3}\;,\;\mathrm{if}\;\hat{{HRV}_{i}}==\gamma \end{array}\right.\;$$

where

(i) The high-risk preictal period criteria in HRV are formulated as
  $$\alpha =\left\{{\left({HRV}_{i}\right)}_{t_{i}}<{\left({HRV}_{i}\right)}_{t_{i+1}}\ll {\left({HRV}_{i}\right)}_{t_{i+2}}\ldots T\right\};$$
(ii) The medium-risk preictal period criteria in HRV are formulated as
  $$\beta =\left\{{\left({HRV}_{i}\right)}_{t_{i}}≅{\left({HRV}_{i}\right)}_{t_{i+1}}<{\left({HRV}_{i}\right)}_{t_{i+2}}\ll {\left({HRV}_{i}\right)}_{t_{i+3}}\ldots T\right\};$$
(iii) The low-risk preictal period criteria in HRV are formulated as
  $$\gamma =\left\{{\left({HRV}_{i}\right)}_{t_{i}}≅{\left({HRV}_{i}\right)}_{t_{i+1}}≅{\left({HRV}_{i}\right)}_{t_{i+2}}{<\left({HRV}_{i}\right)}_{t_{i+3}}\ll {\left({HRV}_{i}\right)}_{t_{i+4}}\ldots T\right\}$$

As modeled in Equation (9), the proposed mechanism fine tunes the preictal probability of every patient (i) with the aim of predicting seizure onset, characterized by the changes in HRV features presented in Table 1. Adaptive learning and fuzzy-inference-based membership functions and rules are generated from the information related to the training data to determine the risk category of the epileptic seizure, and ANFIS-PSO is used to perform the final decision making for the test data. Subsequent to the first-level postprocessing stage by the ANFIS-PSO, in the proposed approach, the localization of the preictal interval is elucidated in the second-level postprocessing stage, rather than modeling preictal duration alone. Accordingly, the optimal interval by which to separate the alarm onset from the seizure onset, Seizure Prediction Horizon (SPH), is modeled for the $P_{k}^{1}$, $P_{k}^{2}$, and $P_{k}^{3}$ patient groups. As stated in the criteria for high-risk preictal periods, observation of sudden or rapid changes in the HRV feature values over time ($t_{i},\;t_{i+1,}t_{i+2},\ldots ,T$) indicate that the patient is at high risk, and is likely to immediately suffer from seizure onset. Similarly, in the medium-risk preictal period criteria, it is stated that the observation of gradual comparative changes in the HRV feature values over time ($t_{i},\;t_{i+1,}t_{i+2},t_{i+3,}\ldots ,T$) indicate that the patient is at medium risk of immediately suffering from seizure onset. As stated in the low-risk preictal period criteria, the observation of comparatively marginal changes in the HRV feature values over time ($t_{i},\;t_{i+1,}t_{i+2},t_{i+3},t_{i+4,}\ldots ,T$) indicates that the patient is at low risk of immediately suffering from seizure onset. As a result, the proposed approach initiates the alarm based on the patient’s risk level to ensure accurate seizure diagnosis. In the proposed system, the alarm onset time is modeled to occur before the seizure onset time by taking the preictal state transition time into consideration. The alarm onset modeling affects the SPH interval when considering the preictal state duration only. SPH is the interval between alarm initialization and seizure occurrence. The segment with two successive much greater HRV features within the time (t), and the preceding segment, with greater HRV features, are localized as the start time of the preictal interval before the SPH. Thus, this work ensures that the patient-specific preictal modeling system supports the preictal detection with the aim of achieving accurate epileptic seizure prediction. The pseudocode for the proposed epileptic siezure prediction system is presented in Algorithm 1.

**Algorithm 1:** Pseudocode of the Proposed Seizure Prediction Methodology**Input:** Epileptic EEG Signals, {S×CH}_Class_**Output:** Seizure Risk Levels {Low, Medium, High}1 **for** all the hospitals and epileptic EEG samples **do**2 **//Preprocessing//**3 **for** all the samples, S **do**4 Apply Butterworth filtering and normalize signals in 1s sliding window length5   **if** a subset of channels obtains comparatively higher accuracy **then**6      Select a subset as optimal channel7   **end if**8 **end for**9 **//FL-based Generalized Model Construction//**10 **for** each hospital/ local EEG dataset **do**11   Segment samples into four different timescales as 1s, 2s, 4s, and 8s12   Validate each segment on each dataset through ROC curve analysis13 **//Bi-timescale approach//**14  **if** a segment has a higher AUC score than others **then**15  Select the first and second higher timescale as optimal timescales of that particular dataset16   **end if**17 **for** each timescale in the selected bi-timescale **do**18   Extract spike sequences using a spiking encoder, i.e., Equation (3)19   Build graphs for the GCNN model20   Implement GCNN until the representation obtained from hidden layers21  **end for**22 Concatenate representations of two timescales at the dense layer of GCNN23 Classify the Preictal and Interictal Classes24 **end for**25 **for** all three local models **do**26   Perform FL-based global aggregation27   Maintain trade-off between the global and local model using Equations (5–8)28 **end for**29 Update local GCNN model based on the global knowledge30 Classify the Preictal and Interictal Classes31 **//Distinct Preictal State Modeling for Seizure Prediction//**32 **for** each hospital/ local EEG dataset **do**33   Retain the preictal probability of each sample34   Obtain ECG-HRV features and Demographic features35     **for** these three inputs **do**36     Apply ANFIS for decision making with the determination of three seizure risk levels using Equation (9)37   **for** the implementation of ANFIS **do**38     Select the best learners using the PSO algorithm39   **end for**40  Determine the seizure risk levels as three classes41  **end for**42 **end for**43 **end for**

### 4.5. Applicability in Real-Time Medical Systems

Recently, medical devices have delivered healthcare services by connecting medical systems with patients and end users [57]. In the medical field, it is crucial to focus on healthcare development from treatment to prevention in order to improve the quality of human life and alleviate the cost of care. Intelligent algorithms can, to a significant extent, ensure highly efficient prevention while decreasing the computational burden and time spent by hospital medical practitioners. With the rise of personalized and preventive care as new treatment modalities, medical instruments or tools can be used as part of the treatment or disease recognition components in hospitals in order to overcome the absence of doctors or minimize the time spent on examining patients’ health data.

The proposed epileptic seizure prediction system simplifies the task of the client (i.e., hospitals or medical centers) by performing a core portion of the system functions on the server, thereby reducing the burden of EEG database maintenance and seizure prediction system development costs on the client side. Interacting with the server on computers via the internet, a client, such as a hospital, can process patient data for epileptic seizure prediction without the requirement of maintenance. Clinicians and medical centers receive intelligence from the prediction system to assist in the monitoring and diagnosis of epilepsy disease, while enabling hospitals to avoid expensive clinical processes. At the hospitals, the application of the proposed system in real-time disease prediction involves testing individual patients’ samples on the designed system through the interface. The hospitals perform their computations virtually using edge resources to overcome the limitations of the resource-constrained client environment. In a nutshell, in the proposed design, the outcome of the preprocessing and classification stages is referred to the coarse-grained personalization model, which acts as a server with a huge database, while the postprocessing stage refers the data to the fine-grained personalization model, which establishes the interaction between the application and the server at the hospitals.

**Figure 8:**
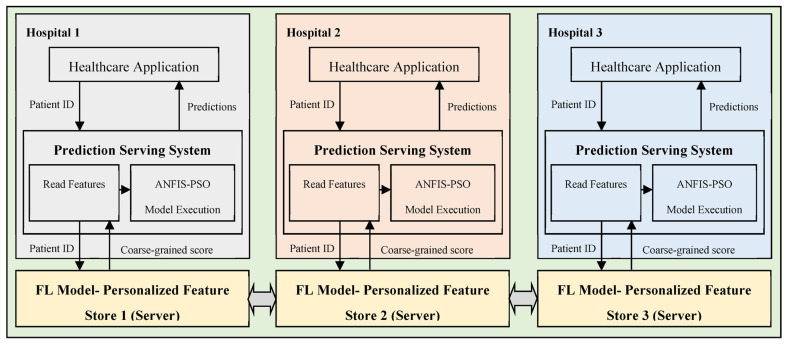
Proposed real-time epileptic seizure prediction method.

Figure 8 depicts the proposed real-time prediction system, which consists of three components. Real-time prediction involves the consitution of a predictive model, which involves the deployment of a coarse-grained personalization model to enable real-time decision making, that is, postprocessing, with the predictive model being built on a huge amount of data. Coarse-grained personalization is a rigorous iterative process that is performed using historical epileptic patient data. Then, to predict epileptic seizures when a continuous stream of patient samples is fed to the system, the predictive model is built, thereby enhancing the end user or client experience. The three major components [58] of real-time predictive analytics include (i) the prediction serving system, (ii) the trained model, and (iii) feature inference. The prediction serving system utilizes a trained learning model to recognize seizures. It provides a prediction outcome for the new input data. In contrast, the trained model and features indicate the data structure, which comprises the weights obtained throughout the training process and data attributes relevant for the prediction.

## 5. Experiments

The effectiveness and performance of our proposed model for early epileptic seizure prediction were assessed, and the performance was compared with baseline models and the results of several works recently published in the literature. The experimental models of early epileptic seizure prediction methods were implemented using the Python programming language. The software environment used for this experimental analysis was Python, running on a 64-bit Ubuntu operating system powered by a 3GHz Intel processing unit and 32GB memory.

Table 3 provides the configuration of the deep learning model of GCNN in the proposed epileptic siezure prediction system. During the implementation of the proposed epileptic seizure prediction algorithm, the numbers of preictal, interictal, and ictal samples for the training set and test set for the CHB-MIT, Bonn, and NSC datasets are mentioned in Table 4.

### 5.1. Datasets

The experiments employed three EEG benchmark datasets, the CHB-MIT scalp EEG dataset, the Bonn EEG dataset, and the New Delhi EEG dataset, to design the FL model for the proposed epileptic seizure prediction method. The datasets were adjusted for the epileptic seizure prediction task by accurately discriminating the preictal class from the interictal or ictal class. The main aim of this work is to perform seizure state prediction by determining the preictal state. Hence, we aim to accurately detect the preictal state on the basis of EEG signals (i.e., the combination of the preictal state with other seizure states like interictal or ictal state). Due to the availability of the preictal and ictal classes only in the benchmark CHB-MIT dataset, an experiment was performed focusing on the discrimination of the preictal and ictal classes, in which the probability of preictal class detection was only addressed when performing postprocessing.

Preprocessed CHB-MIT scalp EEG database: This database was originally gathered through the collaboration of the Children’s Hospital Boston and the Massachusetts Institute of Technology (CHB-MIT), and consists of patients with epileptic seizures that were uncontrollable with medication. This prediction model utilizes the Preprocessed CHB-MIT scalp EEG database [59], containing separate Comma-Separated Value (CSV) preictal and ictal data files for the purposes of performance evaluation. Patients with an adequate number of preictal and ictal samples were selected in order to fit the problem of epileptic seizure prediction. Due to the availability of only the preictal and ictal classes in the preprocessed CHB-MIT dataset, in this work, the preictal state was discriminated from the ictal state when evaluating this dataset.

Bonn EEG dataset: The University of Bonn provides the Bonn EEG dataset [60], comprising five distinct folder subsets. There are 100 single-channel EEG epochs in each file, and they are digitized at a sampling rate of 173.61 Hz using 12-bit A/D resolution. Each EEG epoch contains 4097 samples with a duration of 23.6 s. The Bonn dataset comprises EEG observations from a 100-single-channel system. In this case, a single channel refers to the observations recorded from a single electrode only for each channel. In conclusion, the Bonn dataset has 100 channels that belong to the single-channel recording type. In the Bonn EEG dataset, sets C and D are EEG samples with interictal and preictal states, as described in [5,61].

New Delhi EEG dataset: The Neurology and Sleep Center (NSC) database [62] consists of 1024 EEG samples with a duration of 5.12 s, sampled at 200 Hz. Among the three states—ictal, preictal, and interictal—made publicly available in the NSC dataset, in this work, the preictal and interictal classes are considered for evaluating the seizure prediction algorithm.

Moreover, experiments were conducted on the patients’ clinical records, including their demographic data and ECG-signal-based HRV features, in order to evaluate ANFIS-based decision making in the postprocessing stage of the proposed system. Owing to the lack of ECG data in the benchmark EEG datasets tested in this work, several HRV features for the ECG signals were modeled with reference to [53,54], rather than extracting features from unknown ECG signals. The preprocessing and examination of the ECG signals was outside the research scope of this work. Hence, to prove the influence of the ECG features on decision making with respect to epilepsy, standard ranges of HRV features were synthesized. Furthermore, these three epileptic EEG datasets lack clinical information about each patient. Thus, to test the influence of clinical information on epileptic seizure prediction, patient-specific clinical information was modeled randomly for each dataset.

### 5.2. Performance Metrics

The experiment utilizes the following evaluation metrics: sensitivity, specificity, accuracy, and false positive rate (FPR) to demonstrate the reliability of the proposed model.

Sensitivity: Sensitivity is the ratio between the number of correctly classified preictal samples and the total number of preictal samples to be classified in a particular class. Sensitivity is also known as recall.

(10) $$Sensitivity=\frac{\mathrm{True}\;Positives}{\mathrm{True}\;Positives+\mathrm{False}\;Negatives}$$

Specificity: Specificity is the ratio between the number of correctly classified interictal samples and the total number of interictal samples actually classified.

(11) $$Specificity=\frac{\mathrm{True}\;Negatives}{\mathrm{True}\;Negatives+\mathrm{False}\;Positives}$$

Accuracy: Accuracy measures the overall performance of the model at detecting both the preictal and interictal samples.

(12) $$Accuracy=\frac{\mathrm{True}\;Positives+\mathrm{True}\;Negatives}{\mathrm{True}\;Positives+\mathrm{True}\;Negatives+\mathrm{False}\;Positives+\mathrm{False}\;Negatives}$$

False Positive Rate: FPR measures the number of false positives over the total test period.

(13) $$False\;Positive\;Rate=\frac{\mathrm{False}\;Positives}{\mathrm{False}\;Positives+\mathrm{True}\;Negatives}$$

Area Under the Curve (AUC): AUC quantitatively measures the performance of the learning model at discriminating between true positives and true negatives, with a higher AUC score showing better learning model performance.

Relative Accuracy: To measure the algorithm’s performance even on samples with imbalanced classes, relative accuracy is assessed using the prediction ability of the most frequent class in the samples, referred to as the baseline.

(14) $$Relative\;Accuracy=\left(\frac{Accuracy}{Baseline}-1\right)\times 100$$

where

(15) $$Baseline=\frac{Most\;frequent\;Class}{Total\;Samples}\;$$

where

True Positive: Number of correctly detected preictal samples.

True Negative: Number of correctly detected interictal or ictal samples.

False Positive: Number of incorrectly detected preictal samples.

False Negative: Number of incorrectly detected preictal samples.

### 5.3. Results

In this experimental study, the variation across several baseline models and Existing Epileptic Seizure Prediction (EESP) works was investigated. The baseline models used for comparative purposes were K-Nearest Neighbor (KNN), Decision Tree, Support Vector Machine (SVM), CNN, and LSTM, whereas the EESP works were EESP1 [24], EESP2 [25], and EESP3 [27]. In this experiment, the baseline algorithms were evaluated as classification models for the samples in three benchmark datasets. This section provides the results for the discrimination of the preictal state from the interictal state and the preictal state from the ictal state. In conclusion, the results in the Bonn and NSC datasets were tested on the preictal–interictal samples and the results in the CHB-MIT dataset were tested on the preictal–ictal samples.

Epileptic seizure prediction was realized, and a different anticipation strategy was shown to exist. Thus, the use of a fixed prediction time and the consideration of seizure onset time as a norm become ineffective. This is because the seizure prediction time varies from one patient to another and from one period to another, even for the same epileptic patients. Hence, the testing and evaluation of the seizure prediction algorithm must be conducted on medical cases in real time in order to prove the seizure prediction performance. In conclusion, the solution to the classification problem was evaluated on the basis of the discrimination of preictal state samples from samples of other states to qualify seizure prediction performance in this research work.

In Table 5, the epileptic seizure prediction performance of the proposed method and is compared with the existing models EESP1, EESP2, and EESP3. The evaluated metrics indicate the performance when discriminating between the preictal and interictal classes, exemplifying seizure prediction performance. The proposed method outperformed the models in Table 5 and achieved a prediction performance similar to that of the real-time scenario using the Leave-One-Out Cross Validation (LOOCV) method during training. The comparative baseline models and EESP research used k-fold cross-validation and train–test split to evaluate the CHB-MIT, Bonn, and NSC EEG datasets.

**Figure 9:**
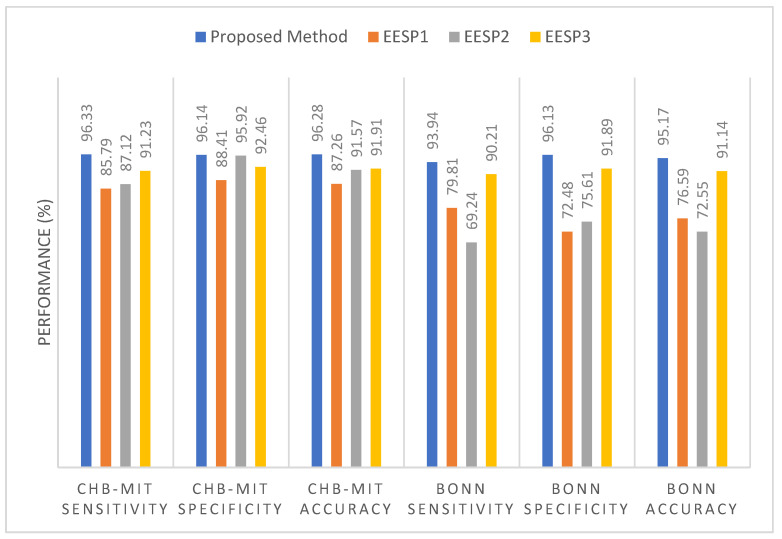
Comparative performance of the proposed method.

Figure 9 illustrates the comparative sensitivity, specificity, and accuracy of the proposed siezure prediction system with the existing works EESP1, EESP2, and EESP3 on both the CHB-MIT and Bonn EEG datasets. The baseline classifiers of the KNN, Decision Tree, and SVM algorithms had a sensitivity of 54.16%, 82.19%, and 89.39%, respectively, when distinguishing the preictal state from the interictal state while evaluating the CHB-MIT dataset. Under the scenario with the same number of patients and samples, our proposed method outperformed the CNN and LSTM deep learning models, with an accuracy that was 11.46% and 6.8% higher, respectively. Compared to EESP1 on the CHB-MIT dataset, the proposed method obtained a 10.54% higher sensitivity and comparatively minimal false positive rate of 0.094. The sensitivity and specificity of our method were comparatively higher than those of other models, and worked while being tested on two EEG datasets, as depicted in Figure 9. Moreover, the proposed approach yielded accuracies of 96.28% and 95.17% on the CHB-MIT and Bonn datasets, respectively, which are 4.37% and 4.03% higher than when using the EESP3 approach.

**Figure 10:**
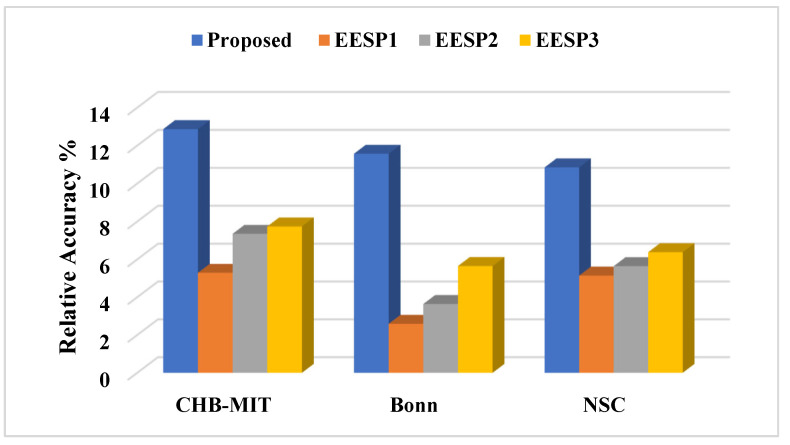
Comparison of relative accuracy.

In Figure 9, the true positive rate and true negative rate were examined in order to validate the performance of the seizure prediction algorithm at detecting the preictal and interictal classes, whereby the assessment of the accuracy metric significantly illustrates the accurate categorization of both epilepsy classes. However, accuracy is not a good metric for assessing the performance of algorithms on the imbalanced data samples in each class. To resolve this, relative accuracy is used to comparatively assess the performance of the seizure prediction algorithm across imbalanced samples with respect to accuracy and baseline values, as illustrated in Figure 10. In the CHB-MIT dataset, the proposed approach obtained a relative accuracy of 12.87% for an accuracy of 96.28%, which is comparatively higher than the existing works EESP1, EESP2, and EESP3.

As presented in Table 5, when using the proposed approach on the Bonn EEG dataset, an average sensitivity of 93.94% and an average FPR of 0.044 were obtained. The overall sensitivity, specificity, and accuracy on the NSC dataset reached 91.11%, 94.24%, and 92.72%, respectively. Thus, the proposed model was able to accurately categorize preictal and interictal seizure states in the Bonn and NSC datasets and preictal and ictal seizure states in the CHB-MIT dataset. It can be seen from Table 5 that better results were obtained using the proposed method than when using the other methods. Even though EESP3 achieved a true negative rate of 95.23%, which is comparatively higher than the proposed method, the accuracy of the proposed prediction model outperformed that of the existing research by improving the true positive rate. All methods were evaluated on three publicly available EEG benchmark datasets, CHB-MIT, Bonn, and NSC, locally and globally using the FL concept. Deciding which model is better for predicting epileptic seizure is an arduous task due to each method needing to be tested using the limited data of different patients on different datasets. Hence, the generalizability of the proposed method is tested without the need for patient-specific clinical data and ECG data, with reference to the proposed method without using the ANFIS-PSO model, that is, the proposed method using SE, GCNN, and FL.

Furthermore, it is evident from Table 6 that the combination of the SE, GCNN, FL, and ANFIS-PSO-based epileptic siezure prediction systems provides higher sensitivity of 96.33% and a higher specificity of 96.14% for the CHB-MIT dataset. Additionally, the sensitivity and specificity achieved on the Bonn and NSC datasets are 93.94% and 96.13%, and 91.11% and 94.24%, respectively. As a result, it can be concluded that the recognition of the preictal state was accurately achieved, through the discrimination either of the ictal state, in the case of the CHB-MIT dataset, or of the interictal state, in case of the the Bonn and NSC datasets. However, there is a marginal variation in the performance measures on different EEG datasets, even when the global model parameters are utilized in the proposed method for updating the local model, due to the variations in time definitions, patients, and epileptic patterns. By examining the performance of the baseline models and existing works presented in Table 6, it is quite apparent that the proposed method yields better results in epileptic seizure prediction on the three different datasets.

Compared to accuracy and specificity, measuring the performance of the detection of the preictal class is extremely important in this research, and sensitivity is a significant measure when validating the epileptic seizure prediction method. Recognition of the preictal state, rather than detecting the interictal and ictal states, is the most crucial process in accurately predicting seizure occurrence, due to the necessity of initializing the warning before the occurrence of a seizure. Table 6 provides a comparison of the performance of the proposed method when using the centralized and federated approaches. In this research, training using a centralized approach consisted of the learning or processing of one EEG dataset at a time, followed by gradient computation and weight updating. Conversely, the federated approach consisted of processing three EEG datasets at once, followed by averaging the weights of the clients.

By introducing the FL in combination with the SE-GCNN model, this work obtained improvements of 1.56% and 0.51% in sensitivity and specificity, respectively, when testing on the CHB-MIT dataset. This enhancement was due to the adoption of the FL model as the global model and the segment-aware generation of the training sample in the proposed system, facilitating the discrimination between the preictal and interictal states. As mentioned in Table 6, the centralized approach had the worst sensitivity, specificity, accuracy, and false positive rate among the proposed models. Consequently, the FL model was adopted for the learning process in the proposed epileptic seizure prediction system. Moreover, the proposed system used the ANFIS model in the postprocessing stage, and the results were influenced by the SE, GCNN, and FL models. As a result, the performance of the proposed model demonstrated an increased sensitivity of 96.33%, an increased specificity of 96.14%, and an increased accuracy of 96.28% for the CHB-MIT dataset, as shown in Table 5. The false positive rate also decreased to 0.032. During postprocessing, the ANFIS-PSO model was tested with different combinations of input data, such as (i) EEG and ECG, (ii) EEG and demographic, and (iii) EEG, ECG, and demographic. The combination of EEG, ECG, and demographic was shown to outperform the other two cases, achieving 91.11% sensitivity and 94.24% specificity on the NSC dataset.

Moreover, Table 7 presents the performance of the proposed method and EESP2 for each of the individual patients or subjects comprising the CHB-MIT dataset. Among all of the patients in the CHB-MIT dataset, few accomplish comparatively best results; for example, patient CHB03 achieved the highest performance, with a sensitivity of 98.27%, an accuracy of 96.09%, and an FPR of 0.071. In the seizure prediction system, the improvement of all of the metrics, including sensitivity, specificity, FPR, and accuracy, was significant. From the analysis presented in Table 7, the average of the specificity results for the different patients using the proposed method is 91.24%. The proposed epileptic siezure prediction system using SE-GCNN and the FL model enforces a minimal false positive rate due to the spiking-sequence-based graph construction and the influence of the local model being updated on the basis of generalized patterns. Moreover, the HRV features of seizure activity accompanied by the EEG-based prediction probability greatly facilitates the achievement of higher sensitivity, with 89.84% being achieved across all patients. The performance presented in Table 7 illustrates that the proposed method ensures stability and maintains the trade-off between the accuracy achieved across all patients and that for a single patient.

**Figure 11:**
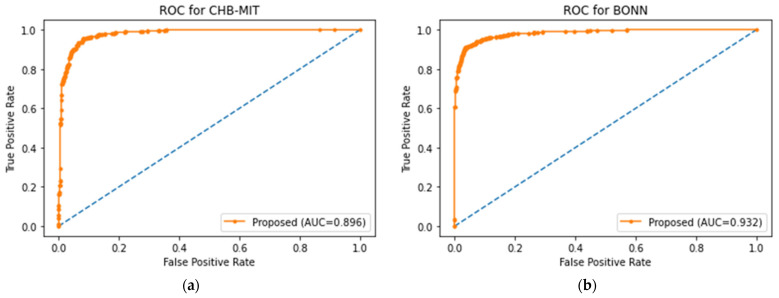

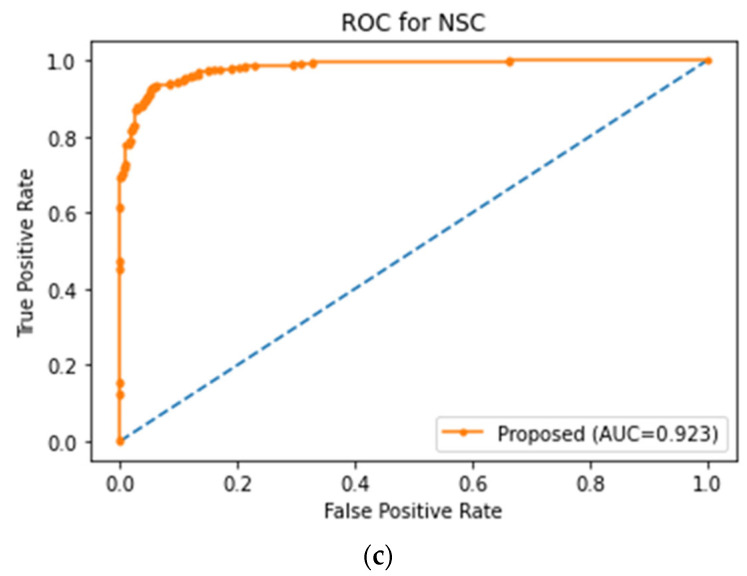
(**a**–**c**) ROC-AUC curve for the proposed epileptic seizure prediction on (**a**) the CHB-MIT dataset, (**b**) the Bonn dataset, and (**c**) the NSC dataset.

The ROC curves and AUC scores of the proposed model when tested on three benchmark datasets are plotted in Figure 11. Figure 11 shows that the proposed model can discriminate the preictal samples from the interictal and ictal samples in all three CHB-MIT, Bonn, and NSC datasets. The implementation of the FL model using a three-tier architecture greatly assists the epileptic seizure prediction system in achieving better AUC scores, with 0.896, 0.932, and 0.923 being achieved on the different EEG datasets by updating the local models with the influence of the global model parameters. As a result, the overall ROC-AUC analysis demonstrates the contribution of the proposed method in terms of ensuring the accurate real-time prediction for all of the generalized seizure patients by implementing FL-assisted coarse-grained personalization and ANFIS-assisted fine-grained personalization modeling.

**Figure 12:**
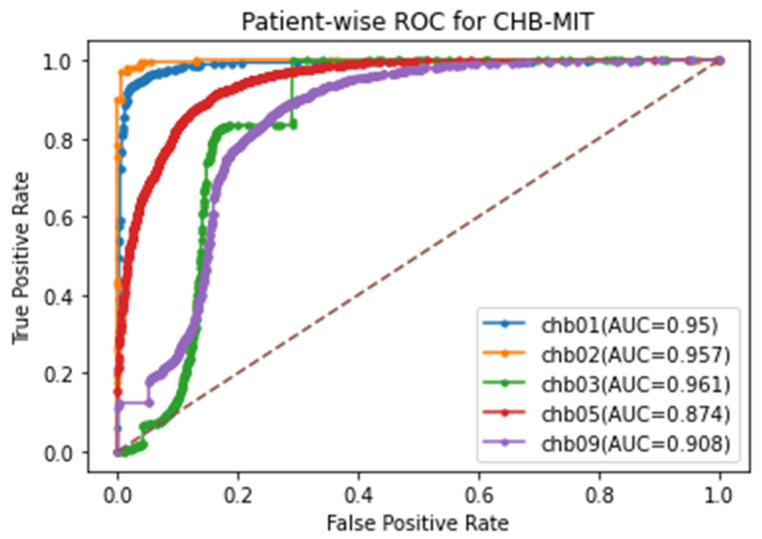
ROC-AUC curve of patient-wise epileptic seizure prediction performance on the CHB-MIT dataset.

Figure 12 illustrates the ROC curve and the AUC score for each patient when tested on the five patients comprising the CHB-MIT dataset. From the analysis of Figure 11 and Figure 12, it can be determined that the proposed epileptic siezure prediction system achieved higher AUC scores, and thus is able to provide accurate seizure predictions for all of the patients, in a patient-specific manner. Among the five epileptic patients tested, the proposed approach was able to accurately predict an epileptic seizure for patient CHB03 with an AUC score of 0.961 on the basis of the discrimination of the preictal state from the ictal samples.

## 6. Conclusions

In this work, a three-tier architecture was designed for FL-based epileptic seizure prediction that addressed the constraints of data scarcity, diversity, and privacy without compromising the accuracy and computational cost by implementing a two-level edge layer. For the modeling of the FL, the global-model-based updating of the local model ensured a balance between model generalization and coarse-grained personalization. The design of a hybrid model with SE and GCNN supported the accurate recognition of the preictal class from the interictal class using segment-aware spike modeling and the bi-timescale approach in coarse-grained personalization. Furthermore, the seizure risk-aware patient personalization was achieved using the ANFIS model, fine-tuning the coarse-grained results obtained from the FL model using the HRV features of ECG signals and patient-specific clinical features. As a result, the proposed system was able to recognize the risk level of the preictal state by examining the preictal probability determined on the basis of the EEG signals. Thus, the experimental results demonstrate that the proposed method outperforms several baseline models, as well as previous research on epileptic seizure prediction, yielding 10.54% and 9.21% higher sensitivity than EESP1 and EESP2, respectively. The proposed method can be extended to design automatic early warning systems with customized seizure prediction horizon times. In addition, the significance of the position of electrodes and channels on the scalp should be considered for the real-time prediction of seizures using deep learning algorithms.

### 6.1. Advantages and Limitations

The proposed approach presents numerous benefits for healthcare services through the potential design of a federated learning-based seizure prediction mechanism. The proposed approach utilizes global epilepsy knowledge through the use of a federated learning model in distributed medical centers. Moreover, the bi-timescale approach and spiking GCNN, along with ANFIS-PSO-based epilepsy state discrimination, in the proposed system enforces the investigation of the temporal as well as spatial relationship of the EEG channel values in the large-scale training dataset, thereby reducing the requirement of the resource capabilities in the medical center.

Nevertheless, the proposed siezure prediction system presents several limitations in terms of providing healthcare services to end users. Firstly, there are variations in EEG data scarcity and hardware resource capabilities among different local hospitals and medical centers; therefore, there is a lack of ability to handle the high dynamics of epileptic EEG signals in the context of sliding window modeling and spike analysis, thereby increasing the classification error. In addition, relying on manual annotations for epilepsy states is critical in real-time seizure prediction, likely increasing number of false positives due to the lack of ability to handle data scarcity with respect to label scarcity.

### 6.2. Future Directions

The proposed method could be extended to design an automatic early warning system with a customized seizure prediction horizon time. In addition, the significance of the position of electrodes and channels on the scalp should be considered for the real-time prediction of seizures using deep learning algorithms. Additionally, future work will include the design of a lightweight real-time seizure forecasting model with the aim of performing time series learning for unlabeled epileptic EEG samples.

## Figures and Tables

**Table 1:** Fuzzy input variables.

No.	Input	Features	Class 1 (Low)	Class 2 (Medium)	Class 3 (High)
1	EEG	Coarse-grained Personalization	0–0.3	0.31–0.6	0.61–1.0
2	ECG (Heart Rate Variability)	Lmax(beats)	198–238	239–245	245–290
		LF/HF	1.3–1.7	1.1–2.1	1.3–2.9
		SDNN (ms)	109–141.6	85.4–165	83.7–159.1
		Mean HR(beats/min)	65.2–76.2	71.6–81.4	72.1–92.5
		pNN50 (%)	6.2–17.6	2.5–8.1	1.6–9.6
3	Patients’ Clinical Records	Genetic	No	Yes	Yes
		Metabolic	No	Yes	Yes
		Number of seizure events	0 < Event < 3	3 ≤ Event ≤ 5	Greater than 5

**Table 2:** HRV Features and its Description.

HRV Features	Description
Lmax	Length of the longest diagonal line
SDNN	Standard deviation of R–R intervals in milliseconds
LF	Spectral density (computed through FFT) of the linear interpolated R–R tachogram between 0.04 and 0.3 Hz (low frequency)
HF	Spectral density (computed through FFT) of the linear interpolated R–R tachogram between 0.3 and 1.3 Hz (high frequency)
Mean HR	Average heart rate
pNN50	Probability of R–R intervals > 50 ms e <−50 ms

**Table 3:** Model parameters.

Learning Parameters	Values
Dropout Rate	0.2
Hidden Units	[16, 16]
Learning Rate	0.01
Activation	GELU, Tanh, Sigmoid
Loss Function	Sparse Categorical Cross-Entropy
Epochs	10
Batch Size	128
Optimizer	Adam

**Table 4:** Samples used for implementation.

Number of Samples	CHB-MIT		Bonn		NSC	
	Preictal	Ictal	Preictal	Interictal	Preictal	Interictal
Training Set	294	312	204	200	149	203
Test Set	756	738	746	750	436	382

**Table 5:** Results of various baseline models and existing models with respect to epileptic seizure prediction.

Comparative Works & Models	CHB-MIT				Bonn				NSC			
	Sen (%)	Spec (%)	Acc (%)	FPR	Sen (%)	Spec (%)	Acc (%)	FPR	Sen (%)	Spec (%)	Acc (%)	FPR
KNN	54.16	77.47	66.15	0.253	46.85	89.26	68.13	0.11	78.10	59.02	68.78	0.412
Decision Tree	82.19	83.70	82.95	0.163	62.28	55.61	58.98	0.443	48.91	59.56	55.01	0.40
SVM	89.39	96.29	92.88	0.037	55.42	80.48	68.04	0.19	75.91	61.20	68.61	0.388
CNN	81.43	88.03	84.82	0.129	66.28	75.61	71.12	0.243	57.66	69.39	63.07	0.316
LSTM	88.63	90.03	89.48	0.105	78.85	77.56	78.25	0.224	69.34	66.12	67.75	0.348
EESP1(EMD+DWT+Decision Tree) [22]	85.79	88.41	87.26	0.126	79.81	72.48	76.59	0.285	76.85	90.10	83.48	0.098
EESP2(LRCN) [23]	87.12	95.92	91.57	0.051	69.24	75.61	72.55	0.243	78.51	56.89	67.82	0.43
EESP3(GAN+CNN+LSTM) [25]	91.23	92.46	91.91	0.084	90.21	91.89	91.14	0.097	89.94	95.23	92.61	0.061
Proposed Method	96.33	96.14	96.28	0.032	93.94	96.13	95.17	0.044	91.11	94.24	92.72	0.057

**Table 6:** Performance evaluation of the proposed method.

Approach	Proposed Method	Input Data	Performance on Benchmark Datasets					
			CHB-MIT		Bonn		NSC	
			Sen (%)	Spec (%)	Sen (%)	Spec (%)	Sen (%)	Spec (%)
Centralized	SE + GCNN	EEG	89.07	91.53	92.27	94.28	88.18	92.22
Federated	SE + GCNN + FL	EEG	90.63	92.04	94.17	95.45	75.14	93.72
Federated	SE + GCNN + FL + ANFIS-PSO	EEG + ECG	91.78	92.31	92.42	94.59	91.08	92.82
Federated	SE + GCNN + FL + ANFIS-PSO	EEG + Patients’ Demographic data	91.10	92.52	93.87	92.28	90.79	93.65
Federated	SE + GCNN + FL + ANFIS-PSO	EEG + ECG + Patients’ Demographic data	96.33	96.14	93.94	96.13	91.11	94.24

**Table 7:** Evaluation of CHB-MIT dataset.

Patient ID	Proposed Method					EESP2 (LRCN) [23]				
	Sen (%)	Spec (%)	Acc (%)	FPR	AUC	Sen (%)	Spec (%)	Acc (%)	FPR	AUC
CHB01	96.33	96.14	96.28	0.032	0.95 ± 0.00	91.32	94.63	93.01	0.067	0.92 ± 0.01
CHB02	85.85	98.31	92.18	0.025	0.957 ± 0.03	80.48	54.76	67.66	0.452	0.521 ± 0.02
CHB03	98.27	93.89	96.09	0.071	0.961 ± 0.01	94.48	96.44	95.47	0.045	0.921 ± 0.03
CHB04	91.41	88.53	90.01	0.124	0.932 ± 0.00	88.98	85.36	87.38	0.147	0.852 ± 0.01
CHB05	94.31	88.84	91.58	0.125	0.874 ± 0.01	87.45	94.29	90.88	0.071	0.891 ± 0.00
CHB06	85.83	89.28	87.61	0.112	0.858 ± 0.05	82.39	89.31	85.94	0.107	0.823 ± 0.02
CHB07	88.16	91.25	89.69	0.086	0.879 ± 0.01	87.16	90.01	88.61	0.098	0.805 ± 0.03
CHB08	82.98	90.21	86.49	0.098	0.839 ± 0.02	78.29	85.61	82.01	0.145	0.826 ± 0.01
CHB09	91.91	88.89	90.51	0.112	0.908 ± 0.00	89.68	90.40	90.04	0.116	0.891 ± 0.01
CHB10	90.05	89.26	89.78	0.113	0.91 ± 0.014	88.35	84.91	86.79	0.156	0.831 ± 0.00
CHB11	92.13	90.34	91.39	0.0953	0.895 ± 0.01	89.63	90.31	89.85	0.097	0.856 ± 0.02
CHB12	89.02	88.91	88.89	0.1112	0.886 ± 0.06	81.26	85.32	83.35	0.149	0.831 ± 0.02
CHB13	89.61	90.27	89.89	0.098	0.901 ± 0.01	88.51	80.32	84.56	0.197	0.795 ± 0.05
CHB14	90.12	85.96	89.01	0.142	0.906 ± 0.03	88.96	82.07	84.62	0.183	0.827 ± 0.02
CHB15	90.29	92.59	91.56	0.0756	0.91 ± 0.00	87.31	90.01	88.75	0.098	0.843 ± 0.01
CHB16	88.20	89.97	89.98	0.112	0.834 ± 0.01	80.12	78.31	80.02	0.218	0.785 ± 0.03
CHB17	87.05	90.28	89.12	0.098	0.865 ± 0.02	76.31	88.04	82.23	0.118	0.814 ± 0.01
CHB18	92.36	91.64	92.05	0.085	0.896 ± 0.01	90.53	89.31	89.87	0.107	0.821 ± 0.02
CHB19	87.89	93.58	91.01	0.0651	0.875 ± 0.1	85.46	90.07	87.82	0.098	0.813 ± 0.01
CHB20	90.21	95.61	92.85	0.0443	0.895 ± 0.02	89.43	92.67	91.12	0.074	0.856 ± 0.03
CHB21	89.31	93.14	91.34	0.069	0.904 ± 0.01	88.91	90.71	89.93	0.094	0.881 ± 0.02
CHB22	90.54	89.04	89.85	0.114	0.912 ± 0.03	89.34	87.05	88.23	0.132	0.851 ± 0.01
CHB23	85.31	90.16	87.89	0.099	0.872 ± 0.02	82.67	89.67	86.29	0.112	0.834 ± 0.01
CHB24	89.19	93.57	91.45	0.0651	0.888 ± 0.01	84.39	91.28	87.91	0.089	0.812 ± 0.02
Average	89.84	91.24	90.69	0.090	0.896 ± 0.02	86.31	87.12	86.76	0.132	0.829 ± 0.02

## Data Availability

The publicly available data set can be found at: CHB-MIT Scalp EEG Database: https://ieee-dataport.org/open-access/preprocessed-chb-mit-scalp-eeg-database (accessed on 5 October 2022). Bonn EEG seizure dataset: https://www.upf.edu/web/ntsa/downloads/-/asset_publisher/xvT6E4pczrBw/content/2001-indications-of-nonlinear-deterministic-and-finite-dimensional-structures-in-time-series-of-brain-electrical-activity-dependence-on-recording-regi? (accessed on 5 October 2022). New Delhi Dataset. Available online: https://www.researchgate.net/publication/308719109_EEG_Epilepsy_Datasets (accessed on 5 October 2022).
